# Increasing Obesity Severity Is Associated With Less Surgical Care in the United States

**DOI:** 10.1002/oby.70240

**Published:** 2026-06-08

**Authors:** Michael Kachmar, Florina Corpodean, Hector J. Garcia Navas, Molly Perkins, Carlos Galvani, Michael W. Cook, Denise M. Danos, David A. Hughes, Philip R. Schauer, Vance L. Albaugh

**Affiliations:** ^1^ Department of Surgery, Morsani College of Medicine University of South Florida Tampa Florida USA; ^2^ Tampa General Hospital Tampa Florida USA; ^3^ Metamor Institute, Pennington Biomedical Research Center at Louisiana State University Baton Rouge Louisiana USA; ^4^ Department of Surgery Louisiana State University Health Sciences Center New Orleans Louisiana USA; ^5^ University Medical Center New Orleans Louisiana USA; ^6^ Department of Behavioral & Community Health Louisiana State University Health Sciences Center New Orleans Louisiana USA; ^7^ Population and Public Health Science Pennington Biomedical Research Center Baton Rouge Louisiana USA; ^8^ Our Lady of the Lake Regional Medical Center Baton Rouge Louisiana USA; ^9^ Baton Rouge General Hospital Baton Rouge Louisiana USA

**Keywords:** body mass index, extreme obesity, NSQIP, obesity, surgery

## Abstract

**Objective:**

Approximately 41% of the US population has obesity, though the prevalence of the highest BMI populations (e.g., ≥ 60 kg/m^2^) is increasing most rapidly. Given the association between obesity and surgical disease burden, we hypothesized that higher BMI groups would account for a growing proportion of operative volume over time. This study examined whether individuals with higher BMI have been receiving proportionally more surgical care over time.

**Methods:**

All multispecialty National Surgical Quality Improvement Program (NSQIP) cases from 2005 to 2022 were analyzed (*n* = 11,634,075). Multinomial logistic regression was performed to analyze trends among BMI categories (< 30.0, 30.0–39.9, 40.0–49.9, 50.0–59.9, 60.0–69.9, and ≥ 70 kg/m^2^), while adjusting for demographics and comorbidities.

**Results:**

Regression models demonstrated decreased odds of patients with BMI ≥ 50 kg/m^2^ undergoing surgery over consecutive years, with increasingly higher BMI groups representing greater declines. Patients with BMI 30.0–39.9 kg/m^2^ demonstrated increased proportional operative volume.

**Conclusions:**

Despite increased growth in higher BMI populations, individuals in these categories undergo fewer surgical operations annually. These findings demonstrate a critical need to understand these inequalities, which are underscored by rising obesity prevalence that may be contributing to poor access to surgical care as well as increased morbidity/mortality of presentation with more advanced disease.

## Study Importance

- Higher BMI obesity prevalence is increasing in the US, which is associated with greater frequency of obesity‐associated diseases that commonly necessitate surgical intervention. However, surgical volume appears to be decreasing in patients with high BMI.
- Reduced surgical intervention for individuals with high BMI or extreme obesity may contribute to increased morbidity and mortality. Understanding the reasons for these potential disparities is critical.

## 1 Introduction

Approximately 41% of US adults have obesity, with the most rapid growth occurring in the highest body mass index (BMI) strata (> 50 and > 60 kg/m^2^), sometimes referred to as “extreme obesity” [1, 2, 3]. Not limited to adults [4], this shift toward extreme obesity has critical implications for health care overall, as extreme obesity is associated with significant comorbidity burden and often requires specialized equipment that is not readily available in most clinical settings [5, 6, 7].

Obesity presents additional challenges for surgical care, however, as obesity is associated with increased burden of multiple common surgical pathologies such as cancer, hernia, biliary disease, gynecologic disease, and osteoarthritis [8, 9, 10]. Aside from challenges in diagnostic work‐up and evaluation, extreme obesity presents multiple unique challenges in the operating room that range from patient positioning, anesthesia management, and increased technical difficulty, as well as a higher prevalence of obesity‐related comorbidities (e.g., diabetes, hypertension, cardiovascular disease) that can complicate perioperative care [11].

Although prior studies have evaluated surgical outcomes in patients with obesity, there is limited literature focusing on patient cohorts with higher BMI (> 50 kg/m^2^). Moreover, no studies have examined how obesity may be affecting overall surgical case volume at a national level. Understanding of the role that obesity plays in surgical case representation is crucial for workforce planning, operating room resource allocation, and perioperative risk stratification. Thus, the current study evaluated the prevalence of all surgical cases across numerous BMI strata (< 30.0, 30.0–39.9, 40.0–49.9, 50.0–59.9, 60.0–69.9, and ≥ 70 kg/m^2^) in the National Surgical Quality Improvement Program (NSQIP, 2005–2022; https://www.facs.org/quality‐programs/data‐and‐registries/acs‐nsqip/). NSQIP is a national accreditation program that includes surgical patient and case data for all surgical specialties. Given the growth in high‐BMI groups, we hypothesized that patients with higher BMI would account for a growing proportion of operative volume over time.

## 2 Methods

NSQIP surgical volume was assessed across BMI strata: < 30.0, 30.0–39.9, 40.0–49.9, 50.0–59.9, 60.0–69.9, and ≥ 70 kg/m^2^ from 2005 through 2022 for all surgery cases in the United States (*n* = 11,634,075). Exclusion criteria included cases with missing demographic data, unknown BMI, unknown surgical specialty, and age < 18 years. The outcome was BMI category and the primary exposure was year.

For maintenance of reproducibility and accuracy, R version 4.4.1 (R Foundation for Statistical Computing, Vienna, Austria) was used for all data manipulations. Opting for R over graphical spreadsheet interfaces allows for a standardized data science approach to NSQIP reporting and mitigates the risk of human error and data loss. The hospitals participating in NSQIP are the source of the data used herein; they have not verified and are not responsible for the statistical validity of the data analysis or the conclusions derived by the authors.

Statistical analyses were performed using SAS/STAT (v. 9.4; SAS Institute Inc., Cary, North Carolina, USA). All statistical tests were two‐sided. Bivariate relationships were assessed via chi‐square tests. Categorical variables were summarized using frequencies and percentages. Age and BMI were summarized as medians and interquartile ranges. Additional analysis with the use of multinomial logistic regression was used to model the odds of case representation per year by BMI, with BMI < 30.0 as the reference group. Multinomial logistic regression was also utilized to evaluate the covariate effects of BMI category across consecutive years on case representation. Multinomial regression was selected as opposed to alternative methods (e.g., ordinal regression) since the primary aim was to compare each BMI category with the base group (BMI < 30) rather than model the cumulative odds of being in a higher BMI category and does not assume a single slope/coefficient across all BMI categories. For data visualization, relative case prevalence over time was visualized using indexed time‐series plots (2005 baseline) with weighted quadratic least‐squares trend lines. Total cases per year, as well as general surgery, gynecologic, cardiac, and thoracic surgery cases, were individually plotted. Models were adjusted for age, race, sex, ASA Class, functional status, dialysis, smoking status, steroid use, diabetes, hypertension, disseminated cancer, heart failure, chronic obstructive pulmonary disease, bleeding disorder, and elective versus emergent surgery. Results from regression models are reported as odds ratios (OR), 95% confidence intervals (CI), and *p* values.

## 3 Results

There were 11,634,075 cases included (57.2% female, 42.8% male), with a median age of 58 and a median BMI of 28.9 kg/m^2^. Most individuals were White (64.3%), followed by other/mixed (17.7%), Black (9.8%), and Hispanic (8.2%). Comorbidities included hypertension (44.39%), diabetes (15.36%), disseminated cancer (2.24%), and heart failure (1.24%). The majority of patients had an ASA Class of 1 and 2 (53.14%), followed by ASA Class 3 (40.58%) and ASA Class 4 and 5 (6.12%) (Table 1).

**TABLE 1:** Baseline case characteristics of NSQIP 2005–2022.

Total, *n* (%)	11,634,075 (100%)	ASA Class, *n* (%)	
Age, median (IQR)	58 (44–69)	ASA Class 1 and 2	6,181,951 (53.14%)
BMI, median (IQR)	28.89 (24.95–34.01)	ASA Class 3	4,708,289 (40.58%)
Sex, *n* (%)		ASA Class 4 and 5	712,093 (6.12%)
Male	4,972,058 (42.75%)	Steroid use, *n* (%)	425,332 (3.66%)
Female	6,656,161 (57.23%)	Disseminated cancer, *n* (%)	260,435 (2.24%)
Race, *n* (%)		Heart failure, *n* (%)	144,340 (1.24%)
White	7,427,772 (64.3%)	Diabetes, *n* (%)	1,786,728 (15.36%)
Black	1,131,826 (9.8%)	Hypertension, *n* (%)	5,164,649 (44.39%)
Hispanic	947,650 (8.2%)	Smoking, *n* (%)	1,960,914 (16.85%)
Other/mixed	2,044,496 (17.7%)		

*Note:* Data from the National Surgical Quality Improvement Program (NSQIP).

Abbreviation: ASA, American Society of Anesthesiologists.

Adjusted multinomial logistic regression with BMI < 30 kg/m^2^ as the reference (Table 2) identified decreased odds of proportional case representation over time for BMI categories: 40.0–49.9 kg/m^2^ (OR: 0.99, *p* < 0.0001); 50.0–59.9 kg/m^2^ (OR: 0.96, *p* < 0.0001); 60.0–69.9 kg/m^2^ (OR: 0.94, *p* < 0.0001); and ≥ 70 kg/m^2^ (OR 0.87, *p* < 0.0001). Patients with BMI 30.0–39.9 were the only group with an increased case volume across consecutive years (OR 1.02, *p* < 0.0001). Similar trends were identified in unadjusted multinomial logistic regression models (Table 3).

**TABLE 2:** Adjusted multinomial logistic regression model of odds ratios for BMI categorization across consecutive years.

BMI category	Odds ratio	Lower 95% CI	Upper 95% CI	*p*
30.0–39.9 kg/m^2^	1.017	1.01705	1.01706	*p* < 0.0001
40.0–49.9 kg/m^2^	0.992	0.99221	0.99222	*p* < 0.0001
50.0–59.9 kg/m^2^	0.961	0.96061	0.96063	*p* < 0.0001
60.0–69.9 kg/m^2^	0.944	0.94414	0.94417	*p* < 0.0001
≥ 70 kg/m^2^	0.871	0.87097	0.87104	*p* < 0.0001

*Note:* Data from the National Surgical Quality Improvement Program. Reference group is BMI < 30 kg/m^2^. Odds ratios adjusted for the following: age, race, sex, ASA Class, functional status, dialysis, smoking status, steroid use, and history of heart failure, chronic obstructive pulmonary disease, and bleeding disorder. Odds ratios represent the change in odds, per 1‐year increase in time, of a surgical case falling into a given BMI category relative to the reference category (BMI < 30.0 kg/m^2^), after adjusting for covariates.

**TABLE 3:** Unadjusted multinomial logistic regression model of odds ratios for BMI categorization across consecutive years.

BMI category	Odds ratio	Lower 95% CI	Upper 95% CI	*p*
30–39.9 kg/m^2^	1.024	1.02365	1.02365	*p* < 0.0001
40–49.9 kg/m^2^	1.003	1.00321	1.00321	*p* < 0.0001
50–59.9 kg/m^2^	0.971	0.97096	0.97097	*p* < 0.0001
60–69.9 kg/m^2^	0.954	0.95405	0.95406	*p* < 0.0001
≥ 70+ kg/m^2^	0.894	0.89416	0.89418	*p* < 0.0001

*Note:* Data from the National Surgical Quality Improvement Program. The reference group is BMI < 30 kg/m^2^. Unadjusted odds ratios represent the change in odds, per 1‐year increase in time, of a surgical case falling into a given BMI category relative to the reference category (BMI < 30.0 kg/m^2^).

Adjusted multinomial logistic regression was also utilized to evaluate the effects of other contributing factors on surgical representation, aside from BMI (Table 4). ASA Class, hypertension, elective surgery status, and history of heart failure were identified as having the strongest positive correlations, particularly in the highest BMI groups (50–59.9, 60–69.9, 70+). ASA Class 4 and BMI ≥ 70 kg/m^2^ had the greatest positive correlations (OR 625, *p* < 0.0001). Similarly, the greatest positive correlations for hypertension and history of heart failure were identified for patients with BMI ≥ 70 kg/m^2^. The strongest correlation  elective surgery status was associated with BMI 50–59.9 kg/m^2^ (OR 1.83, *p* < 0.0001). The strongest negative associations were observed for dialysis, disseminated cancer, and functional dependence. Of these, dialysis and BMI ≥ 70 kg/m^2^ had the strongest negative correlations (OR 0.113, *p* < 0.0001).

**TABLE 4:** Covariate effects from adjusted multinomial logistic regression model of odd ratios for BMI categorization across consecutive years.

Variable	Operation year	Age	Race, Hispanic	Race, other
30–39.9	1.017 (1.017–1.017), *p* < 0.0001	0.977 (0.977–0.977), *p* < 0.0001	0.968 (0.965–0.970), *p* < 0.0001	0.642 (0.640–0.644), *p* < 0.0001
40–49.9	0.992 (0.992–0.992), *p* < 0.0001	0.938 (0.938–0.938), *p* < 0.0001	0.750 (0.749–0.750), *p* < 0.0001	0.519 (0.518–0.520), *p* < 0.0001
50–59.9	0.961 (0.961–0.961), *p* < 0.0001	0.913 (0.913–0.913), *p* < 0.0001	0.623 (0.623–0.623), *p* < 0.0001	0.453 (0.453–0.453), *p* < 0.0001
60–69.9	0.944 (0.944–0.944), *p* < 0.0001	0.908 (0.907–0.908), *p* < 0.0001	0.605 (0.605–0.605), *p* < 0.0001	0.422 (0.422–0.422), *p* < 0.0001
70+	0.871 (0.871–0.871), *p* < 0.0001	0.894 (0.893–0.895), *p* < 0.0001	0.550 (0.550–0.550), *p* < 0.0001	0.357 (0.357–0.357), *p* < 0.0001

Variable	Race, White	ASA Class 2	ASA Class 3	ASA Class 4
30–39.9	0.834 (0.832–0.836), *p* < 0.0001	3.022 (3.014–3.031), *p* < 0.0001	3.868 (3.857–3.879), *p* < 0.0001	2.974 (2.964–2.983), *p* < 0.0001
40–49.9	0.798 (0.794–0.801), *p* < 0.0001	13.124 (13.088–13.160), *p* < 0.0001	76.157 (75.920–76.394), *p* < 0.0001	62.165 (62.118–62.211), *p* < 0.0001
50–59.9	0.745 (0.745–0.746), *p* < 0.0001	10.941 (10.940–10.943), *p* < 0.0001	163.313 (163.271–163.354), *p* < 0.0001	262.449 (262.408–262.489), *p* < 0.0001
60–69.9	0.664 (0.664–0.664), *p* < 0.0001	5.045 (5.044–5.045), *p* < 0.0001	68.328 (68.324–68.331), *p* < 0.0001	173.912 (173.906–173.919), *p* < 0.0001
70+	0.602 (0.602–0.602), *p* < 0.0001	9.574 (9.574–9.574), *p* < 0.0001	143.744 (143.742–143.746), *p* < 0.0001	625.285 (625.276–625.294), *p* < 0.0001

Variable	ASA Class 5	Known bleeding disorder	No diabetes	Insulin‐dependent diabetes
30–39.9	3.140 (3.140–3.140), *p* < 0.0001	0.865 (0.864–0.865), *p* < 0.0001	0.569 (0.568–0.571), *p* < 0.0001	0.988 (0.986–0.990), *p* < 0.0001
40–49.9	64.065 (64.064–64.065), *p* < 0.0001	0.696 (0.696–0.696), *p* < 0.0001	0.481 (0.479–0.482), *p* < 0.0001	1.033 (1.030–1.035), *p* < 0.0001
50–59.9	196.089 (196.089–196.089), *p* < 0.0001	0.650 (0.650–0.650), *p* < 0.0001	0.473 (0.473–0.473), *p* < 0.0001	1.135 (1.135–1.135), *p* < 0.0001
60–69.9	133.987 (133.987–133.987), *p* < 0.0001	0.687 (0.687–0.687), *p* < 0.0001	0.515 (0.515–0.515), *p* < 0.0001	1.231 (1.231–1.231), *p* < 0.0001
70+	547.442 (547.442–547.442), *p* < 0.0001	0.742 (0.742–0.742), *p* < 0.0001	0.531 (0.531–0.531), *p* < 0.0001	1.344 (1.344–1.344), *p* < 0.0001

Variable	Current dialysis	Cancer diagnosis	Elective surgery status	Functional status, partially dependent
30–39.9	0.635 (0.635–0.635), *p* < 0.0001	0.625 (0.625–0.625), *p* < 0.0001	1.509 (1.503–1.514), *p* < 0.0001	0.681 (0.681–0.682), *p* < 0.0001
40–49.9	0.350 (0.350–0.350), *p* < 0.0001	0.333 (0.333–0.333), *p* < 0.0001	1.812 (1.811–1.813), *p* < 0.0001	0.702 (0.702–0.702), *p* < 0.0001
50–59.9	0.186 (0.186–0.186), *p* < 0.0001	0.239 (0.239–0.239), *p* < 0.0001	1.825 (1.825–1.825), *p* < 0.0001	0.912 (0.912–0.912), *p* < 0.0001
60–69.9	0.143 (0.143–0.143), *p* < 0.0001	0.232 (0.232–0.232), *p* < 0.0001	1.525 (1.525–1.525), *p* < 0.0001	1.184 (1.184–1.184), *p* < 0.0001
70+	0.113 (0.113–0.113), *p* < 0.0001	0.211 (0.211–0.211), *p* < 0.0001	1.311 (1.311–1.311), *p* < 0.0001	1.930 (1.930–1.930), *p* < 0.0001

Variable	Functional status, totally dependent	Heart failure history	COPD history	Hypertension
30–39.9	0.474 (0.474–0.474), *p* < 0.0001	1.052 (1.052–1.052), *p* < 0.0001	0.968 (0.967–0.969), *p* < 0.0001	1.809 (1.804–1.815), *p* < 0.0001
40–49.9	0.336 (0.336–0.336), *p* < 0.0001	1.274 (1.274–1.274), *p* < 0.0001	1.000 (1.000–1.001), *p* < 0.0001	2.324 (2.319–2.329), *p* < 0.0001
50–59.9	0.306 (0.306–0.306), *p* < 0.0001	1.385 (1.385–1.385), *p* < 0.0001	1.100 (1.100–1.100), *p* < 0.0001	2.817 (2.816–2.818), *p* < 0.0001
60–69.9	0.412 (0.412–0.412), *p* < 0.0001	1.406 (1.406–1.406), *p* < 0.0001	1.154 (1.154–1.154), *p* < 0.0001	3.067 (3.067–3.067), *p* < 0.0001
70+	0.786 (0.786–0.786), *p* < 0.0001	1.473 (1.473–1.473), *p* < 0.0001	1.246 (1.246–1.246), *p* < 0.0001	3.713 (3.712–3.713), *p* < 0.0001

Variable	Sex, male	Sex, nonbinary	Current smoker	Chronic immunosuppression
30–39.9	0.879 (0.877–0.881), *p* < 0.0001	1.093 (1.093–1.093), *p* < 0.0001	0.696 (0.693–0.698), *p* < 0.0001	0.764 (0.764–0.764), *p* < 0.0001
40–49.9	0.427 (0.427–0.428), *p* < 0.0001	0.743 (0.743–0.743), *p* < 0.0001	0.479 (0.479–0.480), *p* < 0.0001	0.500 (0.499–0.500), *p* < 0.0001
50–59.9	0.350 (0.350–0.350), *p* < 0.0001	0.940 (0.940–0.940), *p* < 0.0001	0.400 (0.400–0.400), *p* < 0.0001	0.385 (0.385–0.385), *p* < 0.0001
60–69.9	0.415 (0.415–0.415), *p* < 0.0001	1.211 (1.211–1.211), *p* < 0.0001	0.390 (0.390–0.390), *p* < 0.0001	0.340 (0.340–0.340), *p* < 0.0001
70+	0.458 (0.458–0.458), *p* < 0.0001	0.000 (0.000–0.000), *p* < 0.0001	0.348 (0.348–0.348), *p* < 0.0001	0.310 (0.310–0.310), *p* < 0.0001

*Note:* Odds ratios are from multinomial logistic regression models with BMI < 30 kg/m^2^ as the reference. Operation year and age were modeled as continuous variables per 1‐year increase. Reference categories for covariates were as follows: race, Black; ASA Class, 1 (no disturbance); diabetes, Insulin‐dependent; sex, female; functional status, Independent. All other variables are binary (bleeding disorder, dialysis, disseminated cancer, elective surgery, heart failure, COPD, hypertension medication use, current smoking, chronic immunosuppression).

Abbreviations: ASA, American Society of Anesthesiologists; COPD, chronic obstructive pulmonary disease.

**FIGURE 1:**
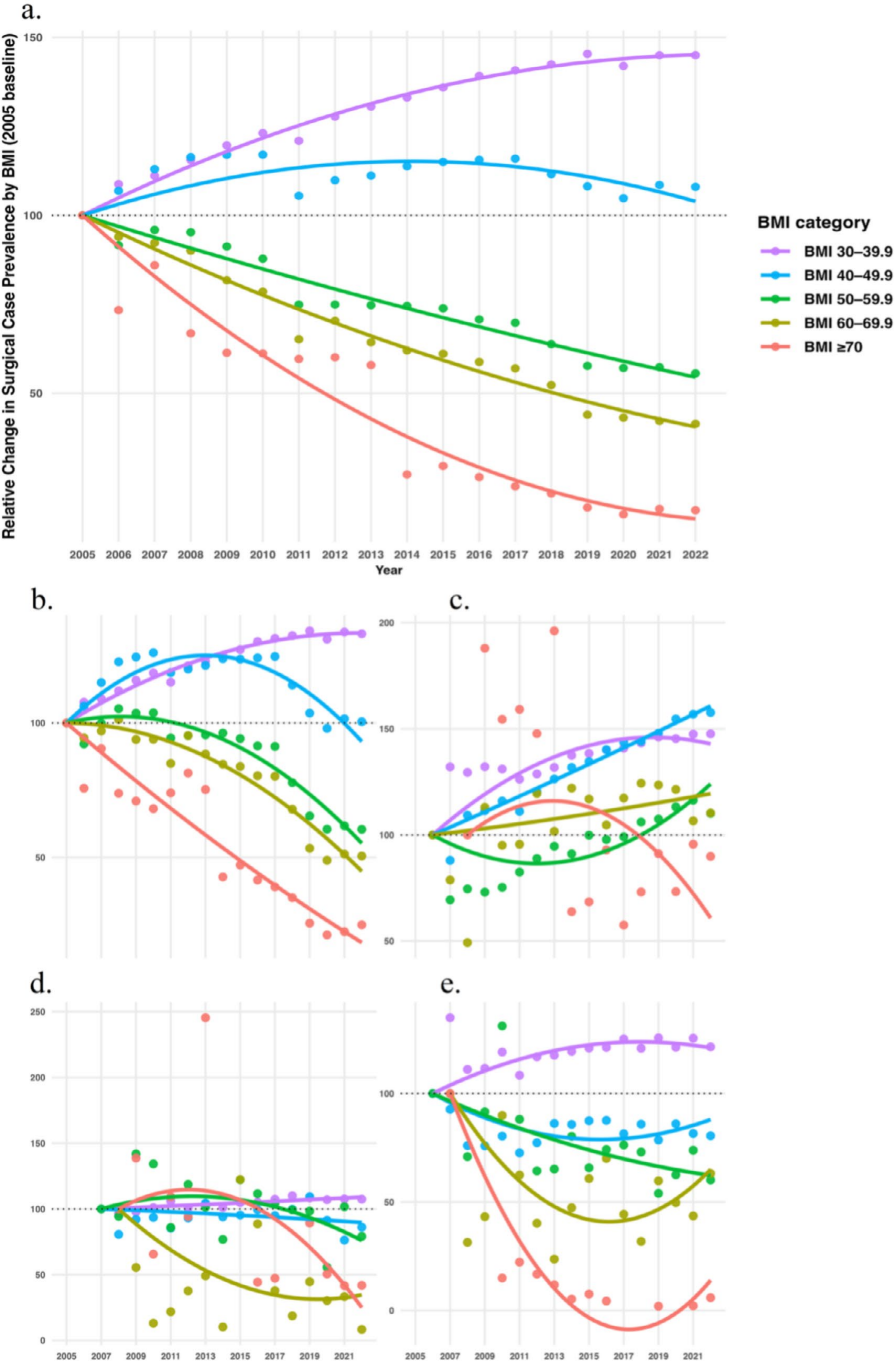
Relative change in surgical case prevalence stratified by BMI, 2005–2022. Curves show the relative change in surgical case prevalence using 2005 as the baseline annual prevalence (y‐axis) over time (x‐axis) stratified by BMI category (defined in the legend by color) for either (a) total cases or (b–e) subspecialty cases. Regression trend lines are weighted quadratic least‐squares fits for each BMI category. Subspecialty cases include: (b) general surgery, (c) gynecology, (d) cardiac surgery, and (e) thoracic surgery. [Color figure can be viewed at wileyonlinelibrary.com]

Total surgical case prevalence trajectories for all surgical specialties are shown in Figure 1a. Individuals with BMI 30–39.9 kg/m^2^ and 40–49.9 kg/m^2^ were the only groups to have an increase in surgical volume from 2005 to 2022. However, individuals with BMI 50–59.9 kg/m^2^, 60–69.9 kg/m^2^, and ≥ 70 kg/m^2^ all had a significant decrease in surgical case prevalence during this time, with the greatest decrease identified in the BMI ≥ 70 kg/m^2^ group.

Surgical case prevalence trajectories by surgical subspecialty are shown in Figure 1b–e. General surgery case trends (Figure 1b) were similar to all surgical case trends, except for the BMI 40–49.9 kg/m^2^ group which had decreased by 2022. Gynecologic case volume (Figure 1c) had an increase in case volume by 2022 for all BMI categories, except for BMI ≥ 70 kg/m^2^ in which it had decreased by this time. Trends for cardiac surgery cases (Figure 1d) revealed a decrease in case volume for all BMI categories except for BMI 30–39.9 kg/m^2^ which had a minimal increase in case volume by 2022. Thoracic surgery case trends (Figure 1e) were comparable with cardiac surgery cases with a decrease in case volume for all BMI categories except for BMI 30.0–39.9 kg/m^2^.

## 4 Discussion

Analysis of > 11 million multispecialty surgical cases from the NSQIP demonstrated a significant decline in surgical volume over time in individuals with BMI ≥ 40 kg/m^2^. This trend worsened with increasing obesity severity, despite the rising prevalence of higher BMI populations nationally over the last two decades [2]. In contrast, patients with BMI 30.0–39.9 kg/m^2^ experienced increased surgical representation, though this accounts for only a fraction of that population's growth [2]. While this relationship between BMI and surgical volume was most pronounced in total and general surgery cases, trends for nonobstetric gynecologic, cardiac, and thoracic procedures were more variable across BMI strata.

Even though overall surgical case volume has not kept pace with increasing prevalence of higher BMI obesity, the reasons underlying these trends are unclear and likely multifactorial. One explanation may be related to increased risk associated with obesity that could affect elective surgical decision‐making. Obesity is associated with longer operative times, increased risk of wound infection, and greater postoperative morbidity when compared to patients with normal BMI [11, 12]. Aside from technical and medical complexity, individuals with higher BMI may also have mobility issues and be unable to seek out health care because of ambulatory status. Despite the observational nature of this study, all these factors affecting access to care would be expected to lead to delayed presentation and worse outcomes, especially with diseases (i.e., cancer) in which late‐stage presentation precludes curative surgical intervention [13].

While overall surgical procedure numbers have decreased over time for higher BMI patients, these changes do not appear to affect all specialties equally. This study used BMI as a surrogate for obesity severity, which is not an indication of adiposity distribution that would be expected to affect some surgical procedures more than others. For example, high‐BMI obesity with significant visceral adiposity will affect an abdominal operation (i.e., ventral hernia repair) much more than an extremity operation (i.e., total knee arthroplasty). The current study shows the clearest relationship between total NSQIP case trends and BMI strata over time, and that data is enriched in general surgery cases which are largely composed of abdominal procedures. Nonobstetric gynecologic also showed trends toward fewer operations with higher BMI, which shares some of the same technical challenges in lower abdominal and pelvic surgery as general surgery [14]. While effects were less robust in cardiac and thoracic surgery subgroups, operations within the chest cavity (e.g., lung resections, cardiac valve repair) may not be as affected by higher BMI as compared to general surgery or nonobstetric gynecology. Moreover, in some cases (i.e., lung resection) obesity may not be associated with worsened short‐term outcomes like other surgical specialties [13, 15]. Finally, many cardiac and thoracic diagnoses (e.g., multi‐vessel coronary artery disease, unstable angina, lung cancer) are not elective and present urgently requiring timely surgical intervention.

Overall, the associations identified in this study raise concerns about potential barriers to surgical care in patients with obesity, including reduced eligibility for elective procedures, inadequate infrastructure to accommodate higher BMI patients, and selection bias in national quality improvement programs. This pattern aligns with a recent national study of subspecialty clinics that demonstrated that over 40% of attempted visits for patients with extreme obesity could not be scheduled due to inadequate equipment or infrastructure, emphasizing logistical constraints as a major determinant for access to care [6]. Similarly, higher BMI obesity is known to be associated with a lower likelihood of routine cancer screening [16, 17], which would also be expected to translate into fewer surgical procedures and more advanced cancer diagnoses. Given the strong association between obesity and surgical disease burden, reduced surgical access may contribute to increased morbidity and more advanced disease at the time of intervention.

The strengths of this study include the use of the largest, national surgical case sample that includes patients with high BMI, an underrepresented population in clinical research. This study is not without limitations, though. NSQIP represents clinical practice, is not randomized, and is prone to selection bias. All cases in NSQIP represent patients who underwent surgery. Nonoperatively managed or individuals that were not surgical candidates are excluded from this data. Furthermore, in some surgical specialties (e.g., orthopedics, transplant), patients may even be deemed ineligible for elective procedures based on BMI [18, 19]. NSQIP does not include all surgical specialties but has increased specialty diversity in recent years; however, the data presented include the specialties present at or near inception of the national quality program. Other limitations include the finite clinical details and restricted generalizability of NSQIP, as well as the retrospective design of this study in which causality cannot be determined.

## 5 Conclusion

Higher BMI or extreme obesity is associated with decreased operative volume, a multifactorial disparity that could be related to perceived risk, health care access, and lack of access to preventive or diagnostic services. Despite the increased prevalence of extreme obesity, these individuals appear to be undergoing fewer operations year after year. There is a critical need to identify the mechanisms underlying these disparities to enable equitable health care access to higher BMI populations.

## Author Contributions

M.K. provided conception and design of the work, was responsible for the acquisition and visualization of the data, and critically revised the manuscript for important intellectual content. M.P. and H.G.N. drafted the initial version of the manuscript and contributed to revisions and data visualization. F.C. and D.A.H. assisted with the conception of the work and revision of drafts. D.M.D. and D.A.H. reviewed the statistical approach and analysis of the data. M.W.C., C.G., and P.R.S. provided critical oversight and revisions of the manuscript. V.L.A. provided critical oversight and conceptualization, contributed to the acquisition of the data, and revised the manuscript. All authors reviewed the manuscript.

## Funding

V.L.A. is supported by DK138261. Data analysis was supported by a grant to the Louisiana Clinical and Translational Science Institute (LACaTS, U54 GM104940). This research was conducted with high‐performance computational resources provided by the Louisiana Optical Network Infrastructure (http://www.loni.org).

## Conflicts of Interest

The authors declare no conflicts of interest.

## Data Availability

The data that support the findings of this study are available from the American College of Surgeons from participating institutions upon reasonable request.
